# The effect of behaviorally anthropomorphic service robots on customers’ variety-seeking behavior: an analytical examination of social presence and decision-making context

**DOI:** 10.3389/frobt.2025.1503622

**Published:** 2025-01-31

**Authors:** Wenchao Liu, Xin Xin, Chenyu Zheng

**Affiliations:** ^1^ School of Business Administration, Jilin University of Finance and Economics, Changchun, China; ^2^ Business School, Jilin Business and Technology College, Changchun, China; ^3^ Strategic Planning Department, China Three Gorges Corporation, Wuhan, China

**Keywords:** service robot, behavioral anthropomorphism, variety-seeking behavior, social presence, consumer decision-making context

## Abstract

The concept of anthropomorphism is crucial in enhancing interactions between service robots and humans, serving as a key consideration in the design of these robots. Nevertheless, the specific mechanisms by which the anthropomorphic traits of service robots influence customer behavioral responses remain inadequately understood. Furthermore, the incorporation of anthropomorphic robotic technology into customer service operational strategies presents a significant challenge for businesses. To explore the underlying mechanisms through which the anthropomorphic characteristics of service robots impact customer acceptance, this study conducted a series of six experiments to empirically test the proposed hypotheses. The empirical findings indicate notable differences in customer switching behaviors and selection quantity metrics, which can be linked to service contexts characterized by varying degrees of behavioral anthropomorphism. Additionally, social presence has been identified as a mediating variable that affects the relationship between the anthropomorphism of service robot behavior and its influence on customer variety-seeking behavior. The situational context of customer decision-making is also found to moderate the relationship between social presence and variety-seeking behavior. Consequently, it is recommended that service organizations implement service robots with diverse anthropomorphic features to enhance customer acquisition, cultivate loyalty, and improve overall marketing effectiveness.

## 1 Introduction

In recent years, the deployment of service robots has become increasingly prevalent across various service sectors, including healthcare, retail, hospitality, and food service (Mende et al., 2019). Service robots, enhanced by artificial intelligence, are system-oriented machines designed to engage in interaction, facilitate communication, and provide customer services in the capacity of AI service agents (Wirtz et al., 2018). Service robots possess the capability to make autonomous decisions by utilizing data obtained from a range of sensors, thereby enabling them to adapt to specific situations (Lu et al., 2019). This trend has enabled service providers to enhance the efficiency and quality of their offerings. Projections indicate that by 2025, humanoid service robots will be further integrated into diverse service experiences (Van Doorn et al., 2017). However, the successful incorporation of robotic technology into customer service remains a significant challenge for many service organizations. A key aspect of service robots is their personification (Zeller and Smith, 2014), which is critical for fostering trust, enhancing social interaction, and encouraging human-robot connections (Li et al., 2010). Consequently, anthropomorphism has emerged as a vital variable in the design of service robots. Nevertheless, determining the optimal application of anthropomorphic features in customer service operations presents a considerable challenge for service enterprises.

The anthropomorphism of service robots’ manifests not only in their physical design but also in their behavioral attributes. The degree of anthropomorphism in service robots can influence consumer responses and their interactions with both employees and other customers within social contexts (Duffy, 2003). Service robots that embody anthropomorphic characteristics and incorporate advanced technologies can offer customers innovative and distinctive service experiences, thereby heightening their engagement and interest (Kozinets et al., 2017), as well as stimulating compensatory consumption and diverse seeking behaviors (Mende et al., 2019). Variety-seeking is a significant characteristic and selection strategy employed by consumers during the acquisition of products (McAlister, 1982; Liu et al., 2015). This behavior reflects consumers’ inclination to seek diversity in their choices of goods and services (Olsen et al., 2016). Currently, there is a paucity of research addressing the marketing implications of service robots, particularly concerning the unique attributes of anthropomorphism and its overall impact on consumers (Huang and Rust, 2018). The extent to which service robots should exhibit anthropomorphic cues, along with the specific consumer and service provider reactions elicited by these robots in commercial settings, has not been thoroughly examined (Belanche et al., 2020). The levels of customer acceptance and interaction modalities represent significant barriers to the adoption of service robots by many organizations (Castelo et al., 2019). While the application and enhancement of anthropomorphic features may facilitate greater acceptance of service robots, the mechanisms through which these features influence customer willingness to accept such technology remain inadequately elucidated. Therefore, to investigate the intrinsic mechanisms by which the anthropomorphic characteristics of service robots affect customer acceptance, it is essential to consider not only the design of the robots but also the characteristics of the customers and the nature of service interactions. This approach will aid in establishing a meaningful research framework and assist practitioners in increasing the likelihood of successfully implementing service robots.

The organization of the subsequent sections of this study is as follows: Section 2 presents a succinct review of the relevant literature. Section 3 outlines the theoretical framework for analysis and articulates the research hypotheses. In Section 4, we detail both the pre-experimental and formal experimental designs. Section 5 discusses the experimental results and their implications. Section 6 addresses the conclusions drawn from the study, acknowledges its limitations, and suggests avenues for future research. To enhance clarity, certain experimental materials are provided in Appendix A.

## 2 Literature review

### 2.1 Anthropomorphism of service robots

Anthropomorphism refers to the inclination of individuals to ascribe human-like characteristics, motivations, intentions, and emotions to both imagined and actual behaviors of non-human entities (Epley, 2018). For robots to facilitate meaningful social interactions with humans, they must exhibit a certain level of personification in either their form or behavior (Duffy, 2003). Anthropomorphism refers to the extent to which consumers ascribe human-like traits to service robots (Blut et al., 2021). Empirical studies suggest that this phenomenon is shaped by consumers’ evaluations of the technology’s human-like attributes, including its physical appearance, nomenclature, facial characteristics, and perceived level of autonomy (Belk et al., 2023). The anthropomorphic design of robots is primarily manifested in two dimensions: appearance and behavior. The anthropomorphism of appearance pertains to the extent to which a robot visually resembles a human. This may include features such as a face or a human-like body, which shape human perceptions of robotic appearance (Aggarwal and McGill, 2007). Walters et al. (2008) categorized service robots into three classifications--mechanical robots, humanoid robots, and android robots--based on the degree of anthropomorphism in their appearance. Among these categories, mechanical robots exhibit the lowest level of anthropomorphic appearance, while android robots display the highest level. Conversely, the anthropomorphism of behavior relates to the extent to which a robot emulates human behavior and is capable of expressing emotions through body movements, vocalizations, and linguistic content during interactions, thereby demonstrating human-like characteristics (Bates, 1994). Kim et al. (2019) differentiated robot behavior into high and low anthropomorphic states based on the degree of anthropomorphism. In a highly anthropomorphic behavioral state, service robots are capable of executing limb movements, nodding, altering body posture, modulating vocal intensity, and conveying emotions through language (Kim et al., 2019). In contrast, a low anthropomorphic behavioral state is characterized by minimal physical movement and emotional expression. In the research undertaken by Lu et al. (2021), the notion of humanlike appearance is confined exclusively to the physical attributes of the service robot, which consequently limits the scope for diverse interpretations of its visual representation. Furthermore, the concept of human-likeness in linguistic style refers to the extent to which the robotic server replicates the behavior of human service staff by employing a literal rather than a figurative mode of communication.

Anthropomorphism plays a significant role in facilitating human acceptance of robots and serves as a fundamental framework for understanding consumer responses to robotic entities (Kim et al., 2019). From a design perspective, robots can elicit immediate human reactions to life-like or social cues without necessitating extensive cognitive processing. Consequently, individuals tend to apply established social interaction patterns and norms, typically reserved for human interactions, to their engagements with robots (Lee et al., 2005). From a human-centered cognitive standpoint, individuals articulate their comprehension of robotic functionality through specific psychological models. The anthropomorphism of both the appearance and behavior of service robots reflects consumers’ social cognition regarding these entities, viewed through the emotional lens of static impressions and the interactive lens of dynamic behavior. Specifically, appearance personification conveys static visual cues, while behavior personification communicates dynamic visual cues to consumers. Research indicates that dynamic visual cues are more effective than static cues in enabling consumers to extract nuanced information (Tidbury et al., 2016). The humanoid design of robots invokes associations with human physical characteristics; thus, the presence of more human-like features enhances the robot’s attractiveness (MacInnis and Folkes, 2017). Furthermore, when robots exhibit outgoing and cheerful behaviors, they are more likely to be accepted and followed by individuals (Goetz et al., 2003). Human-like behavior fosters the attribution of human cognitive processes to robots (MacInnis and Folkes, 2017), suggesting that if robots mimic human actions, individuals may apply their psychological models of human behavior to interpret robotic behavior (Lee et al., 2005). Consequently, consumers may respond to robotic services in a manner analogous to their responses to human services (Kim et al., 2016). Empirical studies suggest that robots possessing human-like traits elicit more pronounced social responses from individuals than those that are less anthropomorphic, potentially affecting perceptions of robotic agency (Kiesler et al., 2008). In particular, there is a positive correlation between the extent of a robot’s human resemblance and the propensity of individuals to ascribe agency to it, as well as to acknowledge its ability to engage in social interactions (Garvey et al., 2023). The anthropomorphism of service robots can engender a heightened sense of immersion for consumers, thereby establishing a social presence within the framework of human-computer interaction. However, an overreliance on anthropomorphic designs, particularly in the case of humanoid robots that closely resemble humans yet remain artificial, may lead to the uncanny valley phenomenon (Mori et al., 2012). This effect can evoke feelings of discomfort or alienation among individuals and may even incite technological biases, such as “speciesism” (Kim et al., 2019).

### 2.2 Social presence

Social presence pertains to the subjective experience of social agents, including humanoid intelligence, and encompasses the sensation of co-presence with others (Biocca et al., 2003). Within the context of robotic service environments, it is defined as the degree to which robotic technology engenders a perception of another social entity’s presence among customers (Van Doorn et al., 2017). The process of anthropomorphism transforms robots from mere emotionless machines into entities capable of forming social and emotional connections with human counterparts. In the realm of human-computer interaction, the absence of a robust sense of social presence reduces the experience of social robots to a mere physical interaction with artificial entities (Fong et al., 2003). The primary objective in the design of social robots within this field is to facilitate a sense of social presence for users during their interactions (Lee et al., 2011). To achieve a genuine social experience in human-computer interaction, social robots must demonstrate a range of behaviors akin to those of real social actors. The level of immersion serves as a critical indicator of social presence, with the capacity to facilitate face-to-face interactions being a significant criterion for the successful establishment of social presence (Catherine et al., 2018). The visual representation of interactive objects can signify their degree of “authenticity” and “humanization,” whereby increased interaction with these objects correlates with heightened social presence. Furthermore, tactile feedback plays a vital role in enhancing the sense of social presence (Catherine et al., 2018).

The concept of social presence exerts a multifaceted influence on consumer attitudes and behaviors related to consumption. According to social impact theory, both the quantity of social participants (referred to as social scale) and the degree of social directness (or proximity) inherent in social presence can significantly affect consumer emotions and self-representation behaviors, ultimately shaping purchasing decisions (Argo et al., 2005). An increase in the scale of social presence is associated with heightened negative emotions and a greater emphasis on impression management among consumers. In response to these negative emotions and the need to maintain a favorable self-image, consumers tend to engage in a broader range of choices during the purchasing process. Furthermore, social presence has been shown to enhance consumers’ perceptions of safety and positively influence their purchasing attitudes (Wang et al., 2015), thereby affecting their behavioral intentions and trust within service contexts. Research conducted by Van Doorn et al. (2017) indicates that social presence impacts consumers’ social cognition and psychological ownership of interactive objects, which subsequently influences consumer satisfaction, loyalty, and repurchase intentions. Additionally, Xie et al. (2019) highlighted the role of social presence in shaping consumer conformity behaviors. Clearly, social presence has emerged as a significant variable affecting consumer decision-making processes. In the context of human-robot interaction, perceived social presence refers to the degree to which individuals perceive a machine as genuinely present (Heerink et al., 2010). van Doorn et al. (2017) proposed the concept of perceived social presence, defining it as “the extent to which machines (such as robots) evoke in consumers the sensation of being in the presence of another social entity.” Social presence holds significant implications in human-robot interaction (HRI), as fostering a robust sense of social presence during these interactions is often considered the primary objective in the design of socially interactive robots (Fong et al., 2003). Social interactions are not solely confined to human beings; rather, technologies have the capacity to replicate human characteristics in both appearance and behavior (Caić et al., 2020). Such interactions between technology and humans are frequently characterized as quasi-social interactions (Carlos et al., 2024).

### 2.3 Variety-seeking

Variety-seeking is a significant characteristic and selection strategy employed by consumers during the acquisition of products (McAlister, 1982; Liu et al., 2015). This behavior reflects consumers’ inclination to seek diversity in their choices of goods and services (Olsen et al., 2016). Academic research offers two primary interpretations of this phenomenon (Sevilla et al., 2019). The first interpretation emphasizes decision-making over time, characterizing diversity seeking as the extent to which consumers switch between various options within the same product category (Mcalister and Pessemier, 1982). For instance, Kahn et al. (1986) posited that the pursuit of diversity represents consumers’ tendency to diverge from their previous brand purchases. Similarly, Sevilla et al. (2019) contended that diversity seeking manifests as a preference for selecting alternatives that differ from prior choices, suggesting that even alternating between two options can elicit change-seeking behavior. The second interpretation focuses on the quantity of distinct items selected within a consumer’s purchasing history or product portfolio (Ratner and Kahn, 2002). Gullo et al. (2019) defined diversity seeking as the ratio of unique product categories acquired within a specific category relative to the total number of items purchased, which can also be conceptualized as a function of the combinations of consumer choices (Sevilla et al., 2019). Consequently, consumer variety-seeking can be articulated as the degree of transition between new and existing products or different brands within the same product category, as well as the quantity and proportion of varied items in consumers’ decision-making processes.

From a social psychology perspective, individuals’ behaviors in seeking diversity can be categorized into two types: true variety-seeking and derived variety-seeking (Mcalister and Pessemier, 1982; Van Trijp et al., 1996). The distinction between these two types lies in the underlying motivation for the observed transformational behavior, which can be either intrinsic or extrinsic in nature (Mcalister and Pessemier, 1982). True variety-seeking is posited to arise from intrinsic motivations, where individuals pursue diversity driven by a desire for change, novelty, or satisfaction with product attributes. In contrast, derived variety-seeking is influenced by external motivations that are not directly linked to an individual’s intrinsic desire for diversity (Mcalister and Pessemier, 1982). Furthermore, research indicates that various factors, including personal characteristics of consumers, product attributes, and environmental or stimulating factors, significantly influence variety-seeking behavior (Gullo et al., 2019; Song and Liu, 2020). Notably, elements such as the composition and size of the product selection set, the complexity of the consumer decision-making context, and the dimensions of the spatial environment are recognized as critical environmental factors that impact consumers’ variety-seeking behavior (Gullo et al., 2019; Levav and Zhu, 2009), garnering increasing attention from scholars in the field.

A service robot functions via an integrated control system that enables it to replicate human behavior, convey emotions, and deliver services to clients (Seo, 2022). Despite the growing utilization of service robots in recent years, the concept of service robots functioning as service representatives remains a novel experience for the majority of customers. Novelty can be defined as the perception of originality that arises from the juxtaposition of current awareness with past experiences (Kim et al., 2021). Customers encounter a unique experience when a service engages their curiosity and satisfies their desire for knowledge within an enhanced context (Sheth et al., 1991). Consequently, novelty can be understood as a divergence in the perception of prior experiences compared to current realities, signifying an experience that is new and unfamiliar, distinct from previous encounters (Albaity and Melhem, 2017). Consumers engage in comparative evaluations of current stimuli against those encountered in the past, recognizing novelty when their present perceptions exceed prior experiences (Lee and Crompton, 1992). Consequently, the pursuit of novelty is intrinsically linked to the quest for diversity and serves as a significant determinant in consumers’ choice motivations and decision-making processes (Petrick, 2002). This phenomenon reflects consumers’ propensity to alter their selections based on recent experiences (Ratner et al., 1999).

## 3 Theoretical analysis and research hypotheses

### 3.1 Examination of the influence of robot behavior anthropomorphism on variety-seeking behavior

Empirical studies have substantiated the influence of anthropomorphism in service robots on customer behavioral intentions. For instance, Mende et al. (2019) demonstrated through experimental research involving humanoid robots that a heightened level of anthropomorphism can elicit discomfort among customers, subsequently prompting compensatory consumer behaviors. Similarly, Liu et al. (2021) demonstrated that, in the realm of tourism service interactions, a higher level of anthropomorphism in service robots is positively associated with customers’ readiness to participate in the value co-creation process. Furthermore, Li and Zheng (2021) highlighted that, from the perspective of appearance anthropomorphism, the extent of anthropomorphism in service robots positively influences customers’ diverse seeking behaviors. Additionally, Zhang and Wang (2022) identified an inverted U-shaped relationship between the level of anthropomorphism in the appearance of service robots and consumers’ willingness to utilize them, indicating that both low and high levels of anthropomorphism may deter usage. Collectively, these studies suggest that the anthropomorphism of service robots can elicit various customer intentions and behavioral responses. However, the current body of research predominantly emphasizes the differential impacts of varying degrees of appearance anthropomorphism, with limited exploration into the effects of differing levels of behavioral anthropomorphism.

Individuals exhibit primary, secondary, and mechanistic variations in their perceptions of the anthropomorphic characteristics and behaviors of service robots. The anthropomorphism of appearance primarily conveys static visual cues to consumers, whereas the anthropomorphism of behavior provides dynamic interactive cues. Distinctions exist between two types of cognition in specific contexts, with corresponding differences in the cognitive regions and mechanisms within the human brain. When individuals assess the anthropomorphic appearance of service robots from a static impression standpoint, they are generally not influenced by the robots’ behaviors. Conversely, when individuals evaluate the anthropomorphic behaviors of service robots through the lens of dynamic interaction, the robots’ actions and the fluidity of human-machine interaction become the focal point of consumer attention, overshadowing the robots’ appearances. Fink (2012) emphasizes that the efficacy of robots in human-computer interaction is more closely tied to their behaviors, with successful behaviors serving as a fundamental aspect of interaction. A high degree of anthropomorphism in behavior facilitates a more natural, effective, and appealing human-computer interaction, fostering a sense of closeness. When robots engage in social behaviors such as making eye contact, nodding, and utilizing arm movements, users exhibit increased attention, trust, and empathy towards these robots (Bartneck et al., 2007). The anthropomorphism of service robot behavior is more likely to evoke a sense of immersion among consumers, thereby establishing a social presence within the context of human-computer interaction; in contrast, merely observing the static appearance of a robot is less effective in generating such a social presence. Furthermore, the anthropomorphic appearance of robots tends to elicit only a brief period of stimulation, primarily during initial encounters. In contrast, the anthropomorphism of robot behavior can engender a lasting sense of social presence that recurs across multiple service interactions. When customers engage with service robots exhibiting anthropomorphic behaviors, they not only experience curiosity and novelty but also perceive the presence of another social entity through interaction and communication, thereby cultivating a robust sense of social presence (Xie et al., 2019). It is evident that merely observing the anthropomorphic appearance of service robots is insufficient to foster a meaningful sense of social presence; rather, it is through the observation and interaction with anthropomorphic behaviors that a genuine sense of social presence can be established.

The extent of anthropomorphism exhibited by service robots significantly influences customers’ ability to accurately conceptualize the robot’s physical form and its activities, thereby impacting the perceived level of social presence. The embodiment theory posits that individuals engage in multi-channel representation and simulate perceptual, kinesthetic, and introspective information pertaining to non-real physical entities during cognitive processing (Wu et al., 2011). When service robots display a higher degree of anthropomorphism, they are capable of executing smoother limb movements, nodding, altering body posture, modulating vocal volume, and articulating emotions through language. In such scenarios, customers are likely to construct a more authentic experience of the robot’s physical presence and its activities, facilitating cognitive processing that activates multi-channel representations and simulates relevant perceptual, action-oriented, and introspective information. This leads to a heightened perception of the robot as a social entity, resulting in an enhanced sense of social presence. Conversely, when the degree of anthropomorphism in robot behavior is minimal, the service robot struggles to perform fluid limb movements, nod, change posture, modulate volume, or convey emotions effectively through speech. In these instances, customers encounter difficulties in realistically conceptualizing the robot’s physical form and engaging with its activities, which hampers cognitive processing. Consequently, they find it challenging to activate multi-channel representations and simulate the necessary perceptual, kinesthetic, and introspective information, leading to a diminished perception of the robot as a social entity. As a result, customers experience a lower sense of social presence in such contexts.

The concept of social presence exerts a multifaceted influence on consumer attitudes and behaviors. According to social influence theory, the quantity of social entities and the degree of social immediacy associated with social presence can significantly affect consumer emotions and self-representation behaviors, which subsequently influence purchasing decisions (Argo et al., 2005). Empirical research has demonstrated that social presence serves as a fundamental prerequisite for various critical consumer behavioral intentions. It has the potential to enhance trust in service contexts, improve perceptions of safety, and positively shape purchasing attitudes (Lee and Shin, 2012). Furthermore, social presence can augment hedonic value and foster favorable consumer attitudes. It also impacts consumers’ social cognition and psychological ownership of interactive objects, thereby influencing their satisfaction, loyalty, and repurchase intentions. Additionally, social presence may affect consumers’ conformity behaviors and their propensity for diverse seeking behaviors. In light of these findings, the following hypotheses are proposed:

Hypothesis 1 Service robots exhibiting higher levels of anthropomorphism in behavior will elicit stronger variety-seeking behaviors from customers compared to those with lower levels of anthropomorphism.

Hypothesis 2 Social presence mediates the relationship between the anthropomorphism of robot behavior and customers’ variety-seeking behaviors.

### 3.2 Examination of the moderating influence of consumer decision-making context

The context in which consumers make decisions plays a crucial role in determining whether their choices are private or public in nature. In public environments, consumers tend to engage in a wider array of behaviors aimed at projecting a distinctive and open personal image (Ariely and Jonathan, 2000). The presence of others creates a clear social consumption scenario, wherein consumer behavior is readily observable. Under these circumstances, consumers’ emotions, cognitions, and behaviors are likely to shift, leading to an increase in diverse seeking behaviors, impulsive purchases, symbolic consumption, and other forms of social consumption (Inman et al., 2009). The Social Facilitation Theory posits that the presence of others can serve as an internal motivator, influencing individuals’ attitudes and behaviors (Kushnir, 1981), and prompting a greater propensity for herd buying decisions (Xie et al., 2019). Ratner and Kahn (2002) conducted a comparative analysis that highlighted the differential effects of private versus public consumption on diversification-seeking behavior, revealing that individuals tend to incorporate more variability into their consumption choices when their actions are subject to public scrutiny. Jinhee et al. (2006) found that consumers are more inclined to pursue diversified options in the presence of others, as their behavior becomes observable, thereby aligning with social norms and the expectations of others. Furthermore, Wang et al. (2020) discovered that the presence of acquaintances diminishes consumers’ diversification-seeking behavior, whereas the presence of strangers enhances it; this indicates that individuals with varying self-conceptions are significantly affected by the social context. Consequently, the number of individuals involved in decision-making processes influences consumers’ diversification-seeking behavior. Based on these findings, the following hypothesis are proposed:

Hypothesis 3 The context of consumption decision-making moderates the effect of social presence on consumers’ variety-seeking behavior.

Hypothesis 3a For consumers engaged in public decision-making, their variety-seeking behavior is significantly heightened when social presence is high compared to when it is low.

Hypothesis 3b There is no significant difference in the influence of social presence on variety-seeking behavior among consumers making private decisions.

**FIGURE 1:**
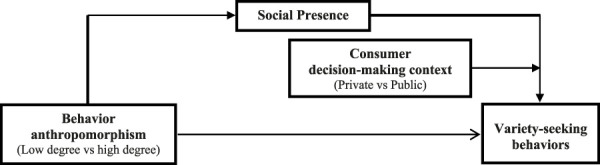
Theoretical model.

In light of the preceding analysis, the theoretical framework is illustrated in Figure 1.

## 4 Research design

### 4.1 Pre-experimental and formal experimental design

The pre-experimental involves the creation and validation of anthropomorphic video materials intended for the examination of service robot behavior. Following a meticulous selection and editing process, two experimental videos were produced to illustrate varying degrees of anthropomorphism in accommodation service robots. These videos were derived from subtitled footage recorded by Kim et al. (2019), which effectively anthropomorphizes robot behavior while controlling for confounding variables. A highly anthropomorphic state refers to service robots that are equipped with the ability to manipulate their limbs, display nodding behaviors, alter their body posture, and modulate their vocal volume, which enables them to convey emotions during verbal exchanges (Kim et al., 2019). Conversely, a low anthropomorphic state indicates that the physical movements and emotional expressions demonstrated by service robots are relatively limited (Kim et al., 2019). For the purposes of this study, the English dialogue in the videos was accurately translated into Chinese, accompanied by corresponding Chinese subtitles, as detailed in the appendix documentation. The videos used for the study are available as Supplementary Material, Supplementary Video 1 is the high anthropomorphic condition and Supplementary Video 2 is the low anthropomorphic condition. Table 1 illustrates the primary distinctions between high-level and low-level anthropomorphic videos. Preliminary testing with a small sample indicated that these two videos successfully differentiate between high and low levels of anthropomorphism in service robots.

**TABLE 1:** The main differences between high-level and low-level anthropomorphic videos.

Category	A video demonstrating high-level of anthropomorphism in behavioral	A video demonstrating low-level of anthropomorphism in behavioral
Body movement	More body movements	Less body movements
Facial expression	Rich facial expressions	Facial expressions are relatively limited
Dialogue	Android robot: Hello handsome! My name is Nadi, I’m glad to serve you! Customer: You look very beautiful Android robot: It’s too much! You also look very handsome! Customer: It’s very happy to chat with you Android robot: Chatting with you is also enjoyable! Customer: How are you feeling today? Android robot: Seeing you, my mood is not ordinary good! Customer: Why do you think so? Android robot: Because you are very friendly! Customer: What else can you do? Android robot: As the hotel front desk, I can provide meticulous assistance to guests! Customer: Great, goodbye! Android robot: Looking forward to seeing you again, handsome!	Android robot: My name is Nadi and I am at the hotel front desk Customer: You look very beautiful Android robot: Thank you! Customer: Nice to chat with you Android robot: Me too Customer: How are you feeling today? Android robot: Sorry, I did not feel anything Customer: Why do you think so? Android robot: I have no perception Customer: What else can you do? Android robot: I am a receptionist and I can assist guests Customer: Bye!

The formal experiment is structured to test hypotheses through a series of six sub-experiments, as outlined in Table 2. Experiments 1a and 1b are designed to validate the primary effect by employing different dependent variable frameworks. Initially, participants will be randomly assigned to an intergroup design based on varying levels of behavior anthropomorphism (low vs. high). They will then assess the anthropomorphism level of the manipulated independent variable. Subsequently, participants will be exposed to distinct robot service scenarios and will engage in a survey regarding their consumption decisions related to hotel accommodations, specifically focusing on their inclination to select hotel dinner packages and the quantity of complimentary fruit juice drinks. Finally, participants will provide demographic information. Experiments 2a and 2b are intended to examine the mediating effect of social presence. In these experiments, interactive service scenarios and catering service scenarios featuring service robots with consistent behavioral characteristics will be developed to enhance the visual stimulation experienced by participants. Following their exposure to interactive video materials showcasing the service robot, participants will evaluate their perceived sense of social presence. After interacting with the catering service scenarios, participants will assess their propensity to switch between dinner meal options and the number of juice drinks they opt to select. Experiments 3a and 3b are focused on verifying the moderating effect of the context in which customers make consumption decisions, with participants being assigned to either public or private decision-making contexts.

**TABLE 2:** Formal experimental design.

Experiments	Experimental purpose	Experimental design	Manipulation of the independent variable	Measurement of the dependent variable	Measurement of other variables
Experiment 1a	The primary impact of validating the anthropomorphic characteristics of service robot behavior on the improvement of customer variety-seeking behavior	The study design consists of a 2 (degree of anthropomorphism in robotic behavior: low versus high) × 2 (degree of variety-seeking: high versus low) factorial arrangement	Participants in the study are exposed to textual descriptions and video representations of various behaviors within the context of accommodation service scenarios	Trends in the conversion rates of dinner package selections within accommodation services	Assess the extent of anthropomorphism in behavioral manifestations
Experiment 1b				The variety of options available among the five distinct flavors of fruit juice beverages	Assess the extent of anthropomorphism in behavioral manifestations
Experiment 2a	Examine the mediating function of social presence in the anthropomorphism of service robot behavior, with the aim of improving customer diversity and conversion rates			Trends in the conversion rates of dinner package selections within accommodation services	Assess the extent of anthropomorphism in behavioral manifestations Seven components of the Social Presence Scale
Experiment 2b	Following the exclusion of the mediating influences of novelty and identity threat, it is essential to assess the mediating function of social presence			The variety of options available among the five distinct flavors of fruit juice beverages	Assess the extent of anthropomorphism in behavioral manifestations Seven components of the Social Presence Scale
Experiment 3a	Examine the moderating influence of the context surrounding customers’ consumption decision-making on their propensity for variety-seeking behavior	The study employs a 2 (anthropomorphism of robotic behavior: low degree versus high degree) × 2 (consumer decision-making context: public versus private) × 2 (variety-seeking degree: high versus low) experimental design		Trends in the conversion rates of dinner package selections within accommodation services	Seven components of the Social Presence Scale
Experiment 3b	Following the modification of the measurement approach for the dependent variable, it is essential to assess the moderating influence of the context surrounding customer consumption decisions on the variety-seeking behavior exhibited by consumers			The variety of options available among the five distinct flavors of fruit juice beverages	Seven components of the Social Presence Scale

Regarding sample selection, both the pilot study and the six formal experiments were conducted using the prominent survey platform in China, SoJump, which operates on a payment basis. The experimental subjects comprised ordinary consumers with diverse backgrounds, including variations in occupation, industry, education, and age, thereby enhancing the internal validity of the experiments. Participation was entirely voluntary, with all participants thoroughly reviewing the experimental instructions prior to commencement, thereby gaining an understanding of the study’s background and objectives, and subsequently consenting to partake in the research. On average, each experimental group consisted of approximately 60 effective participants, with specific demographic information about the sample provided in the analysis results section of each experiment.

The manipulation of the independent variable across the six sub-experiments was informed by the methodology established by Kim et al. (2019). This approach enabled participants to engage with textual descriptions of service scenarios as well as service videos depicting various behaviors, thereby enhancing the consistency of the experimental process. The selection of participants and the execution of the experiments were conducted through reputable online research platforms, incorporating individuals from diverse backgrounds, including variations in occupation, industry, education, and age, which bolstered the internal validity of the study. The six experiments employed two distinct methods for measuring the dependent variable: conversion tendency and selection quantity, thereby augmenting the external validity of the findings.

### 4.2 Variable manipulation and measurement

#### 4.2.1 Manipulation of independent variables

The manipulation of independent variables involves having participants view videos of robotic services that exhibit varying levels of personification within identical service contexts. The experimental scenarios are designed to encompass front desk service, dining service, and complimentary beverage service within the hospitality sector. To mitigate the potential interference of background information, a fictitious restaurant name is employed. The stimulus materials utilized in the experimental design are detailed in the appendix.

#### 4.2.2 Measurement of dependent and mediating variables

To enhance the validity of the experiment, two distinct methodologies were employed to assess customer variety-seeking behavior in this study. The first method focuses on the degree of customer conversion, primarily drawing from the measurement techniques established in the research conducted by Kim and Drolet (2003) and Sevilla et al. (2019). Participants are prompted to respond to two questions pertinent to decision-making regarding dinner set meals, utilizing a seven-point Likert scale. The second method involves measuring consumer choice quantity, inspired by the approaches of Levav and Zhu (2009) and Gullo et al. (2019). Participants are asked to select from five different flavors of fruit juice drinks, and the proportion of participants opting for each specific category is subsequently calculated.

The assessment of social presence is informed by the social presence measurement scale utilized in the research conducted by Lee et al. (2006) within the domain of human-computer interaction. A set of seven questions tailored for hotel service contexts has been developed, including inquiries such as “To what extent do you feel as though you are engaging with an intelligent individual?” and “To what extent do you perceive the presence of a knowledgeable companion?” These questions are evaluated using a seven-point semantic differential scale. Furthermore, to investigate the mediating role of social presence within the framework of the exclusion mechanism, social presence, novelty, and identity threat were concurrently employed as mediating variables in Experiment 2b. Novelty is operationalized through the Perceived Novelty Measurement Scale established by Wells et al. (2010), which comprises three items. The construct of identity threat is derived from the five-item Identity Threat Scale utilized in the studies by Yogeeswaran et al. (2016) and Złotowski et al. (2017) in the field of human-computer interaction, with all constructs being measured on a seven-point Likert scale.

#### 4.2.3 Manipulation of moderating variables

The categorization of consumer decision-making contexts is informed by the studies conducted by Jinhee et al. (2006) and Wang et al. (2020), which delineate two distinct categories: public decision-making contexts and private decision-making contexts. The private decision-making scenario is characterized as follows: “On this day, you are traveling alone on a business trip to M city and planning to stay at Hotel A,” wherein all subsequent consumption decisions are made independently. Conversely, the public decision-making scenario is described as: “On this day, you and a group of five colleagues are on a business trip to M city and intend to stay at Hotel A,” where subsequent consumption decisions are made in the presence of colleagues.

Furthermore, control variables encompass demographic factors of consumers, such as gender, age, and education, as well as the consumers’ level of familiarity with robots.

## 5 Experimental results and discussion

### 5.1 Main effect analysis of the influence of anthropomorphism in service robot behavior on variety-seeking behavior

#### 5.1.1 Examination of variations in customer perception regarding the personification of service robot behavior

Following the viewing of the experimental video materials in Experiment 1a, participants were tasked with assessing the level of anthropomorphism exhibited in the behavior of the service robot. The results of an independent sample t-test revealed that participants’ evaluations of robot video materials characterized by high behavioral anthropomorphism (M_H_ = 5.55, SD = 1.24) were significantly greater than those for materials with low behavioral anthropomorphism (M_L_ = 5.06, SD = 1.46), with t (141) = 2.18, p = 0.031. This finding substantiates the differentiation in robot behavior as perceived by consumers across the two categories of experimental videos.

#### 5.1.2 Examination of the impact of robot behavior anthropomorphism on variety-seeking behavior

**FIGURE 2:**
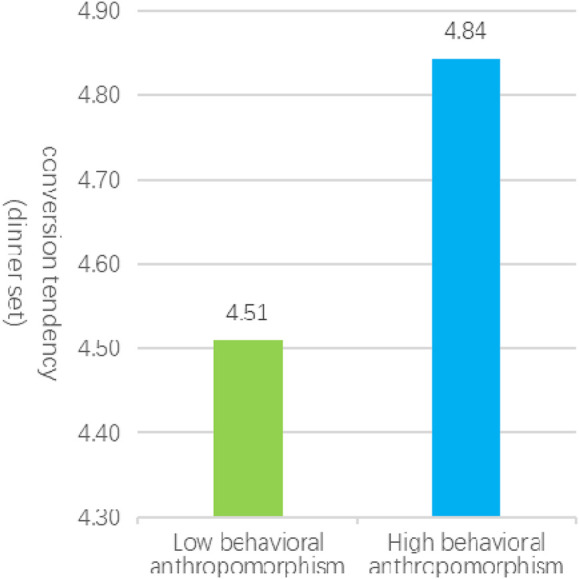
The influence of anthropomorphized behavior on the rate of customer conversion.

In Experiment 1a, a total of 52 valid samples were collected in high behavioral anthropomorphism service scenarios (comprising 25 males and 27 females, with a mean age of 31), while 54 valid samples were obtained in low behavioral anthropomorphism service scenarios (including 28 males and 26 females, with a mean age of 30). An independent samples t-test revealed that the conversion tendency elicited by high behavioral anthropomorphism service scenarios (M_H_ = 4.84, SD = 0.79) was significantly greater than that observed in low behavioral anthropomorphism service scenarios (M_L_ = 4.51, SD = 0.63), with t (104) = 2.30, p = 0.023, as illustrated in Figure 2. These findings provide support for hypothesis Hypothesis 1, indicating that service robots characterized by high levels of behavioral anthropomorphism elicit stronger customer variety-seeking behaviors compared to those with low levels of behavioral anthropomorphism.

**FIGURE 3:**
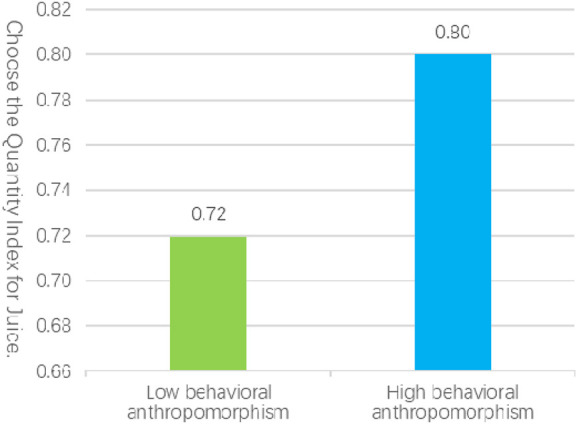
The influence of anthropomorphized behavior on consumer decision-making processes.

In Experiment 1b, a total of 55 valid samples were collected, comprising 25 males and 30 females, with a mean age of 31 years, in high behavioral anthropomorphic service scenarios. In contrast, 53 valid samples were obtained in low behavioral anthropomorphic service scenarios, consisting of 27 males and 26 females, with a mean age of 30 years. An independent samples t-test revealed that the number of choices elicited by high behavioral anthropomorphism service scenarios (M_H_ = 0.80, SD = 0.21) was significantly greater than that elicited by low behavioral anthropomorphism service scenarios (M_L_ = 0.72, SD = 0.22), with t (106) = 2.02, p = 0.046, as illustrated in Figure 3. These findings further substantiate hypothesis Hypothesis 1, indicating that service robots characterized by high levels of behavioral anthropomorphism elicit stronger customer variety-seeking behaviors compared to those with low levels of behavioral anthropomorphism.

### 5.2 Examination of experimental findings regarding the mediating role of social presence

#### 5.2.1 Evaluation of the mediating effect of social presence in isolation

In Experiment 2a, a total of 71 valid samples were collected from participants in high behavioral anthropomorphic service contexts (comprising 37 males and 34 females, with a mean age of 31), and 72 valid samples were obtained from those in low behavioral anthropomorphic service contexts (including 42 males and 30 females, with a mean age of 30). The independent variable was designated as the degree of anthropomorphism in robot behavior, the mediating variable was identified as social presence, and the dependent variable was defined as customer conversion tendency, specifically in relation to dinner set meals. A simple mediation effect analysis was conducted utilizing the Bootstrap method. The findings indicated that the confidence interval values for BootLLCI and BootULCI were (0.0048, 0.1295), which do not encompass zero, thereby confirming the presence of a mediating effect, with an effect size of 0.0581 (refer to Table 2). Furthermore, the confidence interval values for LLCI and ULCI pertaining to the direct effect of the independent variable on the dependent variable were (0.0074, 0.1596), which also exclude zero, signifying a substantial direct effect and partial mediation. Consequently, it can be concluded that Hypothesis 2 is supported, suggesting that social presence serves a partial mediating function in the relationship between the anthropomorphism of robot behavior and customer variety-seeking behavior.

#### 5.2.2 Examination of the mediating role of social presence within the exclusion mechanism

Experiment 2b concurrently investigated social presence, novelty, and identity threat as mediating variables. The study comprised 66 valid responses from participants in high behavioral anthropomorphic service contexts (29 males and 37 females, with a mean age of 30) and 64 valid responses from participants in low behavioral anthropomorphic service contexts (28 males and 36 females, with a mean age of 31). The degree of anthropomorphism in robotic behavior was designated as the independent variable, while social presence, novelty, and identity threat were treated as mediating variables, with the customer choice index serving as the dependent variable. A parallel mediation effect analysis was performed. The findings indicate that, when social presence, novelty, and identity threat are considered as mediators, only social presence exhibited a statistically significant mediating effect (refer to Table 3). The BootLLCI and BootULCI interval values were found to be (0.0036, 0.0321), which excludes zero, thereby confirming the presence of a mediating effect with an effect size of 0.0171. Additionally, the LLCI and ULCI interval values for the direct effect of the independent variable on the dependent variable were (0.0032, 0.0396), also excluding zero, indicating a significant direct effect. This further supports the hypothesis Hypothesis 2. In the context of anthropomorphic robot services, the effects of novelty and identity threat were not statistically significant, thereby negating their roles as mediating mechanisms.

**TABLE 3:** Results of a comparative analysis regarding the mediating effects of social presence.

Dependent variable	Mediating variable	Independent variable	Results of the mediation effect (BootLLCI, BootULCI)	The presence of a mediation effect
The propensity for customer conversion in relation to dinner set meal offerings	Social presence	The extent to which robotic behavior exhibits anthropomorphic characteristics	(0.0048,0.1295)	A mediation effect is present, with a value of 0.0581
Customer Preference Quantity Index (Juice Selection)	Social presence	The extent to which robotic behavior exhibits anthropomorphic characteristics	(0.0036,0.0321)	A mediation effect is present, with a value of 0.0171
	Novelty		(−0.0298,0.0031)	The mediation effect is not present
	Sense of identity threat		(−0.0126,0.0040)	The mediation effect is not present

### 5.3 Examination of the moderating influence of the consumer decision-making context

#### 5.3.1 The moderating influence of consumer decision-making context on conversion tendencies

This study examines the moderating role of consumer decision-making context on the relationship between social presence and variety-seeking behavior, utilizing highly anthropomorphic robotic services as the framework. In Experiment 3a, a total of 71 valid samples were collected from participants in a public decision-making scenario characterized by high personification, while 75 valid samples were obtained from a private decision-making scenario with similar characteristics. The analysis employed customer social presence as the independent variable, customer conversion tendency as the dependent variable, and the context of consumption decision (public versus private) as the moderating variable. A Bootstrap moderation analysis was conducted to assess the moderating effect of the decision-making context. The findings indicated that within the context of highly anthropomorphic robotic services, the moderating effect of consumer decision-making context on the relationship between customer social presence and the degree of conversion in dinner package selection was statistically significant at the 0.10 level (P = 0.0747), with an R^2^ change value of 0.0206. This supports Hypothesis Hypothesis 3, which posits that the consumer decision-making context moderates the influence of customer social presence on variety-seeking behavior.

Further analysis revealed specific regulatory effects. In public decision-making contexts, social presence significantly influenced conversion tendencies related to dinner set meal selection, as indicated by the LLCI and ULCI interval values of (0.1536, 0.6988), which exclude zero, and a P-value of 0.0024. By segmenting customers based on average social presence, independent sample t-tests were performed with customer variety-seeking behavior as the dependent variable. The results demonstrated that customers exhibiting high social presence (M_high presence_ = 5.00, SD = 0.92) displayed a significantly greater willingness to seek diversity compared to those with low social presence (M_low presence_ = 4.43, SD = 0.73), with t (144) = -2.34, p = 0.020. This finding supports Hypothesis Hypothesis 3a, indicating that for consumers engaged in public decision-making, the propensity for variety-seeking is markedly higher among those with elevated social presence.

**FIGURE 4:**
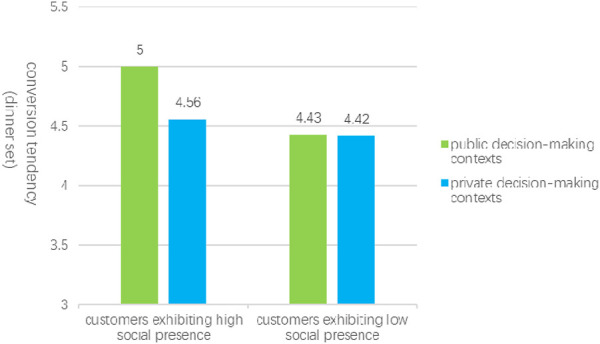
The influence of the consumer decision-making context on the propensity for conversion.

Conversely, in private decision-making contexts, the influence of customer social presence on conversion tendencies (dinner set meal selection) was not statistically significant, as the LLCI and ULCI interval values were (−0.0633, 0.3137), which include zero, and the P-value was 0.1912. In this scenario, customers with high social presence exhibited a willingness to seek diversity (M_high presence_ = 4.56, SD = 0.74), while those with low social presence demonstrated a similar willingness (M_low presence_ = 4.42, SD = 0.81), as illustrated in Figure 4. This suggests that for consumers making private decisions, there is no significant difference in the impact of social presence on variety-seeking behavior, thereby supporting research hypothesis Hypothesis 3b.

In the context of low anthropomorphic robot services, a comparative analysis was performed to examine the differences in the influence of social presence on variety-seeking behaviors among customers in both public and private decision-making environments. In Experiment 3a, the sample comprised 68 valid responses from the low anthropomorphism public decision-making service scenario and 74 valid responses from the low anthropomorphism private decision-making service scenario. The analysis utilized customer social presence as the independent variable, customer conversion tendency as the dependent variable, and the context of customer consumption decisions (private versus public) as the moderating variable, employing Bootstrap moderation analysis. The findings indicated that in the low anthropomorphic robot service scenario, the moderating effect of the customer consumption decision context on the relationship between social presence and customer conversion tendency was not statistically significant, evidenced by an R^2^ change value of 0.0060 and a p-value of 0.3466. Consequently, it is posited that hypothesis Hypothesis 3 is not supported, and therefore, the corresponding hypotheses Hypothesis 3a and Hypothesis 3b do not warrant further investigation.

In summary, within the context of highly anthropomorphic robot services, the consumer decision-making context does moderate the effect of social presence on variety-seeking behaviors. Conversely, in the low anthropomorphic robot service scenario, the moderating effect of the consumer decision-making context is not significant. Thus, it can be concluded that hypotheses Hypothesis 3, Hypothesis 3a, and Hypothesis 3b receive only partial support.

#### 5.3.2 The moderating effect of consumer decision-making context on the number of choices

This study investigates the moderating role of consumer decision-making context on the influence of social presence in highly anthropomorphic robot services, specifically comparing public and private decision-making environments. In Experiment 3b, the sample comprised 68 valid responses from participants in a high personification public decision-making scenario and 70 valid responses from those in a high personification private decision-making scenario. The analysis employed customer social presence as the independent variable, the customer choice index as the dependent variable, and the consumer decision context (public versus private) as the moderating variable. A Bootstrap moderation analysis was performed to assess the moderating effect of the decision-making context on the relationship between social presence and the quantity of choices made by customers. The results indicated a significant moderating effect at the 0.05 level, with an R^2^ change value of 0.0293 and a p-value of 0.0337, thereby supporting hypothesis Hypothesis 3, which posits that the consumption decision context moderates the effect of social presence on variety-seeking behavior.

Further analysis revealed specific moderation effects. In the public decision-making context, the analysis of social presence’s impact on the customer choice index yielded a lower limit confidence interval (LLCI) and an upper limit confidence interval (ULCI) of (0.2365, 0.9426), excluding zero, with a p-value of 0.0012, indicating a significant effect. By categorizing participants into high and low social presence groups based on the mean social presence score, an independent samples t-test was conducted with the customer choice quantity index as the dependent variable. The results demonstrated that the choice quantity index for customers with high social presence (M_H_ = 0.82, SD = 0.21) was significantly greater than that for customers with low social presence (M_L_ = 0.71, SD = 0.23), with t (144) = −2.23, p = 0.027. This supports hypothesis Hypothesis 3a, suggesting that for consumers in public decision-making contexts, variety-seeking behavior is significantly enhanced by high social presence compared to low social presence.

**FIGURE 5:**
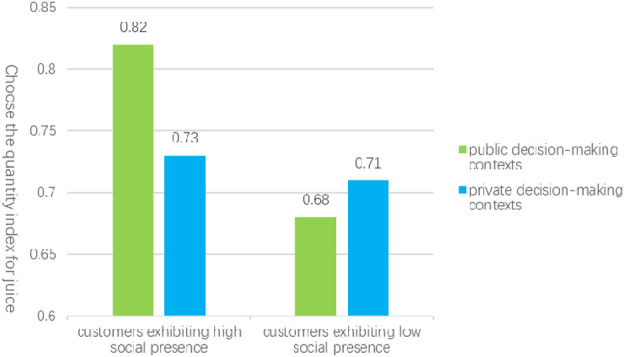
The influence of the consumer decision-making context on the quantity of available options.

Conversely, in private decision-making contexts, the analysis of social presence’s impact on the customer choice index resulted in an LLCI and ULCI of (−0.1896, 0.3827), which includes zero, and a p-value of 0.5057, indicating a lack of significant effect. In this scenario, the choice quantity index for customers with high social presence (M_H_ = 0.73, SD = 0.22) was comparable to that of customers with low social presence (M_L_ = 0.71, SD = 0.21), as illustrated in Figure 5. This finding suggests that for consumers engaged in private decision-making, there is no significant difference in the influence of high versus low social presence on variety-seeking behavior, thereby supporting hypothesis Hypothesis 3b.

This study examines the effects of social presence on variety-seeking behavior among customers in both public and private decision-making contexts, specifically in the context of low anthropomorphic behavior robot services. In Experiment 3b, the sample consisted of 66 valid responses from the low anthropomorphism public decision-making service scenario and 64 valid responses from the low anthropomorphism private decision-making service scenario. The analysis employed social presence as the independent variable, the customer choice index as the dependent variable, and the context of customer consumption decisions (public versus private) as the moderating variable, utilizing Bootstrap moderation analysis. The results indicated that the moderating effect of the quantity index on the relationship between social presence and customer selection of juice beverages was not significant within the low anthropomorphic behavior robot service context. The change in R^2^ was recorded at 0.0101, with a P value of 0.2195. Consequently, it is posited that hypothesis Hypothesis 3 is unsupported, and therefore, the associated hypotheses Hypothesis 3a and Hypothesis 3b do not warrant further investigation.

In conclusion, the findings reaffirm that in scenarios involving highly anthropomorphic behavior robot services, the context of customer consumption decisions significantly moderates the influence of social presence on variety-seeking behavior. Conversely, in low anthropomorphic behavior robot service scenarios, the moderating effect of the decision-making context is negligible. Thus, it can be inferred that hypotheses Hypothesis 3, Hypothesis 3a, and Hypothesis 3b receive only partial support.

### 5.4 Discussion of the findings

The results of the analysis indicate that hypotheses Hypothesis 1 and Hypothesis 2 are substantiated, whereas hypothesis Hypothesis 3 and its sub-hypotheses receive only partial validation. This discrepancy can be attributed to the varying moderating effects of customer consumption decision-making contexts within high and low anthropomorphic behavior robot service scenarios. In scenarios characterized by high anthropomorphic behavior, customer consumption decision contexts significantly moderate the influence of social presence on variety-seeking behavior. Conversely, in low anthropomorphic behavior scenarios, the moderating effect of customer consumption decision-making context is not statistically significant.

In high anthropomorphic behavior robot service scenarios, customers experience an enhanced sense of social presence. This heightened perception arises from the robots’ ability to exhibit more fluid limb movements, nodding, body posture adjustments, volume modulation, and emotional expression through language. Consequently, customers are able to construct a more authentic experience of the robot’s physical form and its activities, engaging in cognitive processing that activates multi-channel representations and simulates perceptual, action-oriented, and introspective information. This process fosters a greater recognition of the robot as a social entity, culminating in an intensified sense of social presence. In such contexts, customers exhibit a higher degree of immersion, allowing them to more vividly perceive the visual representation of the interactive object, engage in more substantial interactions, and clearly observe the collaborative tasks performed by the robot, thereby reinforcing their sense of social presence.

In contrast, low anthropomorphic behavior scenarios present limitations, as the service robots lack the ability to perform smooth limb movements, nod, adjust body posture, or modulate volume, and they struggle to convey emotions through speech. As a result, customers find it challenging to construct a realistic understanding of the robot’s physical form and activities, which impedes their cognitive processing of the experience. This difficulty in activating multi-channel representations and simulating relevant perceptual, action-oriented, and introspective information leads to a diminished recognition of the robot as a social entity, resulting in a lower sense of social presence among customers. Therefore, in high anthropomorphic behavior robot service scenarios, customers generate a more pronounced sense of social presence, with their consumption decision-making context potentially serving as a moderating factor that enhances this experience. Conversely, in low anthropomorphic behavior scenarios, the inherently lower sense of social presence limits the significant regulatory role of customer consumption decision-making contexts. Future research is warranted to explore the specific moderating effects of customer consumption decision-making contexts in greater detail.

## 6 Limitations and future work

This study primarily concentrated on the anthropomorphic characteristics of offline service robots and their associated impacts, excluding the examination of online service robots. In contemporary contexts, a significant number of service robots are deployed across various physical service institutions, yielding favorable marketing outcomes. Conversely, online service robots engage with and assist customers remotely through diverse virtual modalities or through limited anthropomorphism in appearance and behavior. The potential differential effects of the anthropomorphic characteristics of online service robots on customer cognition, responses, and varied seeking behaviors remain unexplored. Future empirical investigations should address the anthropomorphic traits and their implications for online service robots to enhance the breadth of research findings.

Additionally, the research was confined to the accommodation service industry as its focal scenario. Although the study has tested main effects, mediation effects, and moderation effects from multiple levels and perspectives through various experiments, the generalizability of the findings necessitates further validation across different service contexts. Future research should investigate the anthropomorphic characteristics of service robots and their impacts within sectors such as catering, finance, retail, healthcare, and others, particularly concerning their influence on customers’ diverse seeking behaviors, to ascertain the applicability of the conclusions drawn.

Lastly, there are methodological limitations inherent in the research process. Firstly, the experimental component relied solely on humanoid robot videos as stimulus materials, which did not adequately encompass stimuli for both humanoid and mechanical robot experiments. This limitation constrains the generalizability of the anthropomorphic effects associated with service robot behavior. Secondly, the research was conducted exclusively through online experiments, omitting laboratory and field experiments, and the data analysis was restricted to independent sample t-tests, analysis of variance, and Bootstrap mediation and moderation analysis methods. This not only limits the generalizability of the research conclusions but also hinders a comprehensive exploration and analysis of the research data, indicating a need for refinement and enhancement in future studies.

## 7 Conclusion

In recent years, a substantial body of empirical research has emerged concerning service robots, emotional responses, and consumer behavior. Mende et al. (2019) demonstrated that humanoid service robots (SRs) elicit compensatory behaviors, which are influenced by heightened feelings of eeriness. Similarly, van Pinxteren et al. (2019) identified that the anthropomorphism of service robots fosters trust, intention to utilize, and overall enjoyment. Filieri et al. (2022) reported that interactions with robots evoke emotions such as joy, love, surprise, and excitement. Furthermore, Kim et al. (2023) found that engagement with SRs can lead to an increased propensity for unethical consumer behaviors, attributed to diminished anticipatory guilt. Additionally, Pitardi et al. (2024) concluded that interactions with SRs yield more favorable attitudinal and behavioral responses from consumers. Building upon these findings, our research aims to further investigate the relationship between the degree of anthropomorphism in service robots and the diverse seeking behaviors exhibited by customers, thereby contributing to the existing literature on the influence of service robots.

This study elucidates the mechanisms through which the anthropomorphic behavior of service robots influences customers’ variety-seeking behaviors, employing a series of six experiments to investigate the main, mediating, and moderating effects within this framework. The findings yield several key conclusions: Firstly, the extent of anthropomorphism exhibited by service robots significantly affects customers’ variety-seeking behaviors. The results indicate that scenarios involving service robots with a high degree of behavioral anthropomorphism elicit greater customer conversion tendencies and selection indices compared to analogous scenarios characterized by low levels of behavioral anthropomorphism. Secondly, social presence serves as a mediating factor in the relationship between the anthropomorphism of service robot behavior and customers’ variety-seeking behaviors. When the anthropomorphism of service robot behavior was treated as the independent variable, with social presence as the sole mediating variable and customer conversion tendency as the dependent variable, the analysis confirmed the existence of a complete mediating effect attributable to social presence. Additionally, an examination of the parallel mediating effects of social presence, novelty, and identity threat revealed that only social presence exhibited a significant mediating effect, while the effects of novelty and identity threat were found to be non-significant. Thirdly, the context of customer consumption decisions serves as a moderating factor influencing the relationship between social presence and variety-seeking behavior. The effects of this moderating variable differ in scenarios involving high versus low anthropomorphic behavior in robotic services. Consequently, as the consumer decision-making context transitions from a private to a public setting, the effect of social presence on consumers’ tendencies to seek diversity in their choices also undergoes a transformation.

## Figures and Tables

**FIGURE A1:**
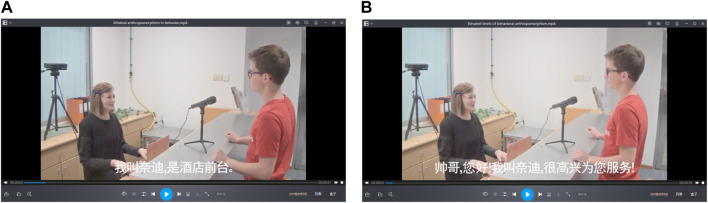
A visual representation captured from the video utilized in the anthropomorphism study concerning the behavior of service robots. **(A)** Minimal anthropomorphism in behavior **(B)** Elevated levels of behavioral anthropomorphism.

## Data Availability

The raw data supporting the conclusions of this article will be made available by the authors, without undue reservation.

## Appendix

### B Stimulus materials for experimental design

The stimulus materials developed for the front desk service experiment are as follows: Hotel A is characterized as a large, stylish, and efficient establishment situated in the vibrant area of M City, offering comprehensive amenities and a variety of room types. On the day in question, you are on a business trip to M City and intend to stay at Hotel A for a duration of five nights. Upon your arrival at the hotel front desk, you observe that the front desk staff resembles a service robot. This piques your curiosity, prompting a brief interaction with the robot. The simulated scenario of your communication with the service robot is depicted in the accompanying video.

The stimulus material designed for the dinner service experiment is as follows: Upon arriving at the hotel and missing the designated dinner time, you request that the hotel front desk arrange for dinner delivery to your room. The service robot, Nadi, informs you that there are currently two dinner packages available for selection, both priced similarly. One package consists of a familiar dinner option, while the other presents a new offering.

The design of the stimulus material for the complimentary drink service experiment is outlined as follows: During check-in, the service robot Nadi informs you that the hotel provides each guest with a complimentary bottle of juice every night. Having stayed for a total of five nights, you have the option to receive all five bottles of juice at once. Nadi will introduce you to the five available flavors of fruit juice. At this juncture, what choice would you make?

## References

[B1] Aggarwal P. McGill A. L. (2007). Is that car smiling at me? Schema congruity as a basis for evaluating anthropomorphized products. J. Consumer Res. 34 (4), 468–479. 10.1086/518544

[B2] Albaity M. Melhem S. B. (2017). Novelty seeking, image, and loyalty-the mediating role of satisfaction and moderating role of length of stay: international tourists’ perspective. Tour. Manage Persp 23 (July), 30–37. 10.1016/j.tmp.2017.04.001

[B3] Argo J. Dahl W. D. Manchanda V. R. (2005). The influence of a mere social presence in a retail context. J. Consumer Res. 32 (9), 207–212. 10.1086/432230

[B4] Ariely D. Jonathan L. (2000). Sequential choice in group settings: taking the road less traveled and less enjoyed. J. Consumer Res. 27 (3), 279–290. 10.1086/317585

[B5] Bartneck C. Kanda T. Mubin O. Mahmud A. A. (2007). The perception of animacy and intelligence based on a robot’s embodiment. IEEE-RAS Int. Conf. Humanoid Robots. 10.1109/ICHR.2007.4813884

[B6] Bates J. (1994). The role of emotion in believable agents. Commun. ACM 37 (7), 122–125. 10.1145/176789.176803

[B7] Belanche D. Casaló L. V. Flavián C. Schepers J. (2020). Service robot implementation: a theoretical framework and research agenda. Serv. Industries J. 40 (3-4), 203–225. 10.1080/02642069.2019.1672666

[B8] Belk R. Belanche D. Flavián C. (2023). Key concepts in artificial intelligence and technologies 4.0 in services. Serv. Bus. 17 (1), 1–9. 10.1007/s11628-023-00528-w

[B9] Biocca F. Harms C. Burgoon J. K. (2003). Toward a more robust theory and measure of social presence: review and suggested criteria. PRESENCE Virtual Augmented Real. 12 (5), 456–480. 10.1162/105474603322761270

[B10] Blut M. Wang C. Wünderlich N. V. Brock C. (2021). Understanding anthropomorphism in service provision: a meta-analysis of physical robots, chatbots, and other AI. J. Acad. Mark. Sci. 49, 632–658. 10.1007/s11747-020-00762-y

[B79] Čaić M. Avelino J. Mahr D. Odekerken-Schröder G. Bernardino A. (2020). Robotic versus human coaches for active aging: An automated social presence perspective. Int. J. Soc. Robot. 12, 867–882. 10.1007/s12369-018-0507-2

[B11] Carlos F. Belk R. W. Belanche D. Luis V. C. (2024). Automated social presence in AI: avoiding consumer psychological tensions to improve service value. J. Bus. Res. 2024, 175. 10.1016/j.jbusres.2024.114545

[B12] Castelo N. Schmitt B. Sarvary M. (2019). Human or robot? Consumer responses to radical cognitive enhancement products. J. Assoc. Consumer Res. 4 (3), 217–230. 10.1086/703462

[B13] Catherine S. O. Jeremy N. B. Gregory F. W. (2018). A systematic review of social presence: definition, antecedents, and implications. Front. Robotics AI 5 (10), 1–35. 10.3389/frobt.2018.00114 PMC7805699 33500993

[B14] Duffy B. R. (2003). Anthropomorphism and the social robot. Robotics Aut. Syst. 42 (3-4), 177–190. 10.1016/S0921-8890(02)00374-3

[B15] Epley N. (2018). A mind like mine: the exceptionally ordinary underpinnings of anthropomorphism. J. Assoc. Consumer Res. 3 (4), 591–598. 10.1086/699516

[B17] Filieri R. Lin Z. Li Y. Lu X. Yang X. (2022). Customer emotions in service robot encounters: a hybrid machine-human intelligence approach. J. Serv. Res. 25 (4), 614–629. 10.1177/10946705221103937

[B18] Fink J. (2012). Anthropomorphism and human likeness in the design of robots and human-robot interaction. Int. Conf. Soc. Robotics, 199–208. 10.1007/978-3-642-34103-8_20

[B19] Fong T. Nourbakhsh I. Dautenhahn K. (2003). A survey of socially interactive robots. Robotics Aut. Syst. 42 (3/4), 143–166. 10.1016/S0921-8890(02)00372-X

[B20] Garvey A. M. Kim T. Duhachek A. (2023). Bad news? Send an AI. Good news? Send a human. J. Mark. 87 (1), 10–25. 10.1177/00222429211066972

[B22] Goetz J. Kiesler S. Powers A. (2003). Matching robot appearance and behavior to tasks to improve human–robot cooperation. 12th IEEE Int. Workshop Robot Hum. Interact. Commun. 2003 Proc. ROMAN 2003, 55–60. 10.1109/roman.2003.1251796

[B23] Gullo K. Berger J. Etkin J. Bollinger B. (2019). Does time of day affect variety-seeking? J. Consumer Res. 46 (1), 20–35. 10.1093/jcr/ucy061

[B24] Heerink M. Krose B. Evers V. Wielinga B. (2010). Assessing acceptance of assistive social agent technology by older adults: the almere model. Int. J. Soc. Robot. 2 (4), 361–375. 10.1007/s12369-010-0068-5

[B25] Huang M. H. Rust R. T. (2018). Artificial intelligence in service. J. Serv. Res. 21 (2), 155–172. 10.1177/1094670517752459

[B26] Inman J. J. Russell S. W. Rosellina F. (2009). The interplay among category characteristics, customer characteristics, and customer activities on in-store decision making. J. Mark. 73 (5), 19–29. 10.1509/jmkg.73.5.19

[B27] Jinhee C. Kim B. K. Choi I. Yi Y. (2006). Variety-seeking tendency in choice for others: interpersonal and intrapersonal causes. J. Consumer Res. 32 (4), 590–595. 10.1086/500490

[B28] Kahn B. E. Kalwani M. U. Morrison D. G. (1986). Measuring variety-seeking and reinforcement behaviors using panel data. J. Mark. Res. 23 (May), 89–100. 10.2307/3151656

[B29] Kiesler S. Powers A. Fussell S. R. Torrey C. (2008). Anthropomorphic interactions with a robot and robot–like agent. Soc. Cogn. 26 (2), 169–181. 10.1521/soco.2008.26.2.169

[B30] Kim H. S. Drolet A. (2003). Choice and self-expression: a cultural analysis of variety-seeking. J. Personality Soc. Psychol. 85 (2), 373–382. 10.1037/0022-3514.85.2.373 12916577

[B31] Kim S. Chen P. Zhang K. (2016). Anthropomorphized helpers undermine autonomy and enjoyment in computer games. J. Consumer Res. 43 (2), 282–302. 10.1093/jcr/ucw016

[B32] Kim S. H. Yoo S. R. Jeon H. M. (2021). The role of experiential value, novelty, and satisfaction in robot barista coffee shop in South Korea: COVID-19 crisis and beyond. Serv. Bus. 16 (3), 771–790. 10.1007/s11628-021-00467-4

[B33] Kim S. Y. Schmitt B. H. Thalmann N. M. (2019). Eliza in the uncanny valley: anthropomorphizing consumer robots increases their perceived warmth but decreases liking. Mark. Lett. 30 (3), 1–12. 10.1007/s11002-019-09485-9

[B34] Kim T. Lee H. Kim M. Y. Kim S. Duhachek A. (2023). AI increases unethical consumer behavior due to reduced anticipatory guilt. J. Acad. Mark. Sci. 51 (4), 785–801. 10.1007/s11747-021-00832-9

[B35] Kozinets R. Patterson A. Ashman R. (2017). Networks of desire: how technology increases our passion to consume. J. Consumer Res. 43 (5), 659–682. 10.1093/jcr/ucw061

[B36] Kushnir T. (1981). The status of arousal in recent social facilitation literature: a review and evaluation of assumptions implied by the current research model. Soc. Behav. and Personality Int. J. 9 (2), 185–191. 10.2224/sbp.1981.9.2.185

[B37] Lee E. J. Shin S. Y. (2012). When the medium is the message how transportability moderates the effects of politicians' twitter communication. Commun. Res. 41 (8), 1088–1110. 10.1177/0093650212466407

[B38] Lee K. M. Jeong E. J. Park N. Ryu S. (2011). Effects of interactivity in educational games: a mediating role of social presence on learning outcomes. Int. J. Human-Computer Interact. 27 (7), 620–633. 10.1080/10447318.2011.555302

[B39] Lee K. M. Peng W. Jin S. A. Yan C. (2006). Can robots manifest personality? An empirical test of personality recognition, social responses, and social presence in human–robot interaction. J. Commun. 56 (4), 754–772. 10.1111/j.1460-2466.2006.00318.x

[B40] Lee S. L. Lau Y. M. Kiesler S. Chiu C. Y. (2005). Human mental models of humanoid robots. Proc. 2005 IEEE Int. Conf. Robotics Automation, ICRA, 2767–2772. 10.1109/ROBOT.2005.1570532

[B41] Lee T. H. Crompton J. (1992). Measuring novelty seeking in tourism. Ann. Tour. Res. 19 (4), 732–751. 10.1016/0160-7383(92)90064-v

[B42] Levav J. Zhu R. (2009). Seeking freedom through variety. J. Consumer Res. 36 (4), 600–610. 10.1086/599556

[B43] Li D. J. Rau P. L. Ye L. (2010). A cross-cultural study: effect of robot appearance and task. Int. J. Soc. Robotics 2 (2), 175–186. 10.1007/s12369-010-0056-9

[B44] Li X. G. Zheng C. Y. (2021). Research on the mechanism of the influence of anthropomorphism degree of service robots on diversified customer behavior. Industrial Technol. Econ. (5), 130–137.

[B45] Liu L. Zheng Y. H. Chen R. (2015). Is it better to choose more-- the impact of selection set size on customer diversification seeking. J. Psychol. 47 (1), 66–78. 10.3724/sp.j.1041.2015.00066

[B46] Liu X. Xie L. S. Li D. M. (2021). Research on the impact of anthropomorphism of tourism service robots on customer value co-creation willingness. Tour. J. 36 (6), 13–26.

[B47] Lu L. Cai R. Y. Dogan G. (2019). Developing and validating a service robot integration willingness scale. Int. J. Hosp. Manag. 80, 36–51. 10.1016/j.ijhm.2019.01.005

[B48] Lu L. Pei Z. Zhang T. T. (2021). Leveraging “human-likeness” of robotic service at restaurants. Int. J. Hosp. Manag. 94, 102823. 10.1016/j.ijhm.2020.102823

[B49] MacInnis D. J. Folkes V. S. (2017). Humanizing brands: when brands seem to be like me, part of me, and in a relationship with me. J. Consumer Psychol. 27 (3), 355–374. 10.1016/j.jcps.2016.12.003

[B50] McAlister L. (1982). A dynamic attribute satiation model of variety-seeking behavior. J. Consumer Res. 9 (9), 141–150. 10.1086/208907

[B51] Mcalister L. Pessemier E. (1982). Variety seeking behavior: an interdisciplinary review. J. Consum. Res. 9 (3), 311. 10.1086/208926

[B53] Mende M. Scott M. L. Van Doorn J. Grewal D. Shanks I. (2019). Service robots rising: how humanoid robots influence service experiences and elicit compensatory consumer responses. J. Mark. Res. 56 (4), 535–556. 10.1177/0022243718822827

[B54] Mori M. MacDorman K. F. Kageki N. (2012). The uncanny valley [from the field]. IEEE Robotics and Automation Mag. 19 (2), 98–100. 10.1109/MRA.2012.2192811

[B55] Olsen S. O. Tudoran A. A. Honkanen P. Verplanken B. (2016). Differences and similarities between impulse buying and variety seeking: a personality-based perspective. Psychol. and Mark. 33 (1), 36–47. 10.1002/mar.20853

[B56] Petrick J. F. (2002). Development of a multi-dimensional scale for measuring the perceived value of a ser vice. J. Leis. Res. 34 (2), 119–134. 10.1080/00222216.2002.11949965

[B57] Pitardi V. Wirtz J. Paluch S. Kunz W. H. (2024). Metaperception benefits of service robots in uncomfortable service encounters. Tour. Manag. 105, 104939. 10.1016/j.tourman.2024.104939

[B58] Ratner R. K. Kahn B. E. (2002). The impact of private versus public consumption on variety-seeking behavior. J. Consumer Res. 29 (2), 246–257. 10.1086/341574

[B59] Ratner R. K. Kahn B. E. Kahneman D. (1999). Choosing less-preferred experiences for the sake of variety. J. Consum. Res. 26 (1), 1–15. 10.1086/209547

[B60] Seo S. (2022). When female (male) robot is talking to me: effect of service robots’gender and anthropomorphism on customer satisfaction. Int. J. Hosp. Manag. 102, 103166. 10.1016/j.ijhm.2022.103166

[B61] Sevilla J. Lu J. Kahn B. E. (2019). Variety seeking, satiation, and maximizing enjoyment over time. J. Consumer Psychol. 29 (1), 89–103. 10.1002/jcpy.1068

[B62] Sheth J. N. Newman B. I. Gross B. L. (1991). Why we buy what we buy: a theory of consumption values. J. Bus. Res. 22 (2), 159–170. 10.1016/0148-2963(91)90050-8

[B63] Song X. F. Liu F. J. (2020). Review of literature on customer variety-seeking behavior. New Econ. (1), 30–34.

[B64] Tidbury L. O'Connor A. Wuerger S. (2016). A systematic comparison of static and dynamic cues for depth perception. Vis. Psychophys. Physiological Opt. 57 (2), 1–9. 10.1167/iovs.15-18104 27379579

[B65] Van Doorn J. Mende M. Noble S. M. Hulland J. Ostrom A. L. Grewal D. (2017). Domo arigato Mr. Roboto: emergence of automated social presence in organizational frontlines and customers’ service experiences. J. Serv. Res. 20 (1), 43–58. 10.1177/1094670516679272

[B66] Van Pinxteren M. M. Wetzels R. W. Rüger J. Pluymaekers M. Wetzels M. (2019). Trust in humanoid robots: implications for services marketing. J. Serv. Mark. 33 (4), 507–518. 10.1108/jsm-01-2018-0045

[B67] Van Trijp H. C. Hoyer W. D. Inman J. J. (1996). Why switch? Product category: level explanations for true variety-seeking behavior. J. Mark. Res. 33 (3), 281–292. 10.2307/3152125

[B68] Walters M. L. Syrdal D. S. Dautenhahn K. te Boekhorst R. Koay K. L. (2008). Avoiding the uncanny valley: robot appearance, personality and consistency of behavior in an attention-seeking home scenario for a robot companion. Aut. Robots 24 (2), 159–178. 10.1007/s10514-007-9058-3

[B69] Wang S. Lilienfeld S. O. Rochat P. (2015). The uncanny valley: existence and explanations. Rev. General Psychol. 19 (4), 393–407. 10.1037/gpr0000056

[B70] Wang Y. Liu P. Sun G. H. (2020). The influence of various types of individuals-whether acquaintances or strangers-on consumers' variety-seeking behavior. J. Central Univ. Finance Econ. (4), 91–97.

[B71] Wells J. D. Campbell D. E. Valacich J. S. Featherman M. (2010). The effect of perceived novelty on the adoption of information technology innovations: a risk/reward perspective. Decis. Sci. 41 (4), 813–843. 10.1111/j.1540-5915.2010.00292.x

[B72] Wirtz J. Patterson P. G. Kunz W. H. Gruber T. Lu V. N. Paluch S. (2018). Brave new world: service robots in the frontline. J. Serv. Manag. 29 (5), 907–931. 10.1108/JOSM-04-2018-0119

[B73] Wu Q. P. Feng C. Chen B. B. (2011). An examination of social cognition research within the context of the embodied framework. Adv. Psychol. Sci. 19 (3), 336–345.

[B74] Xie Y. Li C. Q. Gao P. Liu Y. (2019). The influence and mechanism of social presence on online conformity consumption in live streaming marketing: a behavioral and neurophysiological perspective. Prog. Psychol. Sci. 27 (6), 990–1004. 10.3724/sp.j.1042.2019.00990

[B75] Yogeeswaran K. Złotowski J. Livingstone M. Bartneck C. Sumioka H. Ishiguro H. (2016). The interactive effects of robot anthropomorphism and robot ability on perceived threat and support for robotics research. J. Human-Robot Interact. 5 (2), 29–47. 10.5898/jhri.5.2.yogeeswaran

[B76] Zeller F. Smith D. H. (2014). What a hitchhiking robot can teach us about automated coworkers. Harv. Bus. Rev.

[B77] Zhang Y. Wang Y. G. (2022). Research on the impact mechanism of anthropomorphism of service robots on consumer willingness to use - the moderating effect of social class. Foreign Econ. Manag. 44 (3), 3–18.

[B78] Złotowskia J. Yogeeswaranb K. Bartneck C. (2017). Can we control it? Autonomous robots threaten human identity, uniqueness, safety, and resources. Int. J. Hum. - Comput. Stud. 100, 48–54. 10.1016/j.ijhcs.2016.12.008

